# Information and communication technology demands at work: the association with job strain, effort-reward imbalance and self-rated health in different socio-economic strata

**DOI:** 10.1007/s00420-016-1140-8

**Published:** 2016-05-19

**Authors:** Magdalena Stadin, Maria Nordin, Anders Broström, Linda L. Magnusson Hanson, Hugo Westerlund, Eleonor I. Fransson

**Affiliations:** 1 School of Health and Welfare, Jönköping University, P.O. Box 1026, 551 11 Jönköping, Sweden; 2 Stress Research Institute, Stockholm University, Stockholm, Sweden; 3 Department of Psychology, Umeå University, Umeå, Sweden; 4 Department of Clinical Neurophysiology, Linköping University Hospital, Linköping, Sweden; 5 Institute of Environmental Medicine, Karolinska Institutet, Stockholm, Sweden

**Keywords:** ICT demands, Job strain, Effort-reward imbalance, Self-rated health, Socio-economic status

## Abstract

**Purpose:**

The use of information and communication technology (ICT) is common in modern working life. ICT demands may give rise to experience of work-related stress. Knowledge about ICT demands in relation to other types of work-related stress and to self-rated health is limited. Consequently, the aim of this study was to examine the association between ICT demands and two types of work-related stress [job strain and effort-reward imbalance (ERI)] and to evaluate the association between these work-related stress measures and self-rated health, in general and in different SES strata.

**Methods:**

This study is based on cross-sectional data from the Swedish Longitudinal Occupational Survey of Health collected in 2014, from 14,873 gainfully employed people. ICT demands, job strain, ERI and self-rated health were analysed as the main measures. Sex, age, SES, lifestyle factors and BMI were used as covariates.

**Results:**

ICT demands correlated significantly with the dimensions of the job strain and ERI models, especially with the demands (*r* = 0.42; *p* < 0.01) and effort (*r* = 0.51; *p* < 0.01) dimensions. ICT demands were associated with suboptimal self-rated health, also after adjustment for age, sex, SES, lifestyle and BMI (OR 1.49 [95 % CI 1.36–1.63]), but job strain (OR 1.93 [95 % CI 1.74–2.14) and ERI (OR 2.15 [95 % CI 1.95–2.35]) showed somewhat stronger associations with suboptimal self-rated health.

**Conclusion:**

ICT demands are common among people with intermediate and high SES and associated with job strain, ERI and suboptimal self-rated health. ICT demands should thus be acknowledged as a potential stressor of work-related stress in modern working life.

## Background

Stress caused by psychosocial working conditions is an issue of occupational and public health. Commonly used models of work-related stress are the demands-control (job strain) model, measuring the relationship between work demands and control (Karasek and Theorell 1990), and the effort-reward imbalance (ERI) model, measuring the relationship between work effort and reward (Siegrist 1996). Many employees are exposed to work-related stress defined as job strain or ERI, which has been found to be associated with suboptimal self-rated health (Hoven and Siegrist 2013), cardiovascular diseases (Kivimaki et al. 2012; Nyberg et al. 2013), type 2 diabetes (Novak et al. 2013; Nyberg et al. 2014) and depression (Theorell et al. 2014) among others. This implies increased rates of sickness absence, entailing considerable societal costs (The Swedish Social Insurance Agency 2014).

The commonly used scales to measure job strain and ERI cover traditional work stressors well, but may not fully reflect potential stressors in modern working life, such as stressors associated with information and communication technology (ICT). The use of ICT at work may improve work productivity (Cardona et al. 2013; Chesley 2014), but may also be associated with work intensity in terms of faster work, more interruptions and increased multitasking (Chesley 2014). ICT also enables work during leisure time (Johansson-Hidén et al. 2003). According to the Swedish Work Environment Authority, 36 % of gainfully employed people were working outside the contractual working hours at least once a week in 2013 (the Swedish Work Environment Authority 2014). This may affect the possibility of recuperation, which may increase the risk of negative health outcomes in the long run (Chesley 2014). As a result of the intensive use of ICT at work, concepts such as ICT demands (or similar concepts such as “telepressure” or “technostress”) have been introduced (Barber and Santuzzi 2015; Chesley 2014; Day et al. 2012; Johansson-Hidén et al. 2003; Stenfors et al. 2013).

ICT demands are characterised by potential ICT-related stressors in the work environment such as frequent interruptions by the telephone and emails, claims to give immediate answers to emails and telephone calls that require a lot of work, and computers and other ICT equipment that fail to work properly (Johansson-Hidén et al. 2003; Stenfors et al. 2013). Hence, ICT demands may give rise to experience of work-related stress in modern working life, but the knowledge about how ICT demands are related to established work-related stress models is limited.

ICT demands have been observed to be associated with distress and cognitive complaints in terms of difficulties with concentration, memory, decision-making and ability to think clearly (Chesley 2014; Day et al. 2012; Stenfors et al. 2013). However, knowledge about ICT demands and its relation with other health indicators is far from sufficient. For instance, the association between ICT demands and suboptimal self-rated health has not been examined, to the best of our knowledge. Self-rated health reflects the general state of health, and can be used as a predictor of future health status and mortality, and is thus a central complement to clinical health measures (Singh-Manoux et al. 2007; Stenholm et al. 2014; Waller et al. 2015) Exploring further how ICT demands affect different health indicators such as self-rated health would add important information to the field of occupational and public health.

In previous studies measuring work-related stress as well as self-rated health, social gradients have been observed. Job strain, ERI and physical job demands are more prevalent in people from low SES, and psychosocial work demands are more prevalent in people from high SES (Hammig and Bauer 2013; Hoven and Siegrist 2013; Hoven et al. 2015). Concerning self-rated health, suboptimal self-rated health has been observed to be more prevalent in people from low SES (Alvarez-Galvez et al. 2013; Hammig and Bauer 2013; Hoven and Siegrist 2013; Kjellsson 2013; Toivanen 2011). However, the knowledge about the prevalence of ICT demands in different SES strata and the association with suboptimal self-rated health is very limited.

The aim of this study was to examine the association between ICT demands and two types of work-related stress [job strain and effort-reward imbalance (ERI)] and to evaluate the association between these work-related stress measures and self-rated health among gainfully employed people, in general. Due to previous observations of social gradients in the psychosocial work environment and in self-rated health (Alvarez-Galvez et al. 2013; Hoven and Siegrist 2013), an additional aim was to evaluate the association between work-related stress measures and self-rated health, separately in different SES strata.

## Methods

### Material and participants

**Fig. 1:**
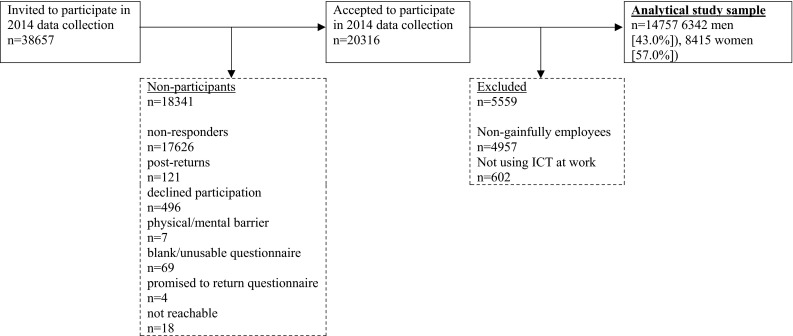
Analytical study sample. *Note* “Non-gainfully employees” refers to people who are working less than 30 % paid work or not at all, e.g. unemployed, on long-term sick leave, homeworkers and retired; “Not using ICT at work” refers to people that answered “I do not have access to this at work” on at least three items in the ICT demands scale

Data from the Swedish Longitudinal Occupational Survey of Health (SLOSH) were used. SLOSH aims to examine connections between work participation, work environment, social situation and health/well-being (Magnusson Hanson et al. 2008, 2015; Statistics Sweden 2014). The study started in 2006 when previous respondents of the 2003 the Swedish Work Environment Survey (SWES) were invited to participate in a follow-up survey. This population has been followed up biennially thereafter, and participants also from SWES 2005–2011 have successively been invited to respond to questionnaires. In the present study, cross-sectional data collected in 2014 were used (Fig. 1). In the 2014 data collection, 38,657 SWES participants were invited, of which some had been asked earlier and some asked for the first time to respond to SLOSH follow-up questionnaires. In total, 20,316 persons (response rate 52.6 %) responded. Out of those who were invited to participate, men, younger people and people with low SES were somewhat less likely to respond (Statistics Sweden 2014). Out of the 20,316, those who were working less than 30 % in paid work or not at all (e.g. retired or on long-term sick leave), or reported that they did not use ICT in their work, were excluded, leaving 14,757 gainfully employed men (6342 [43.0 %]) and women (8415 [57.0 %]), 20–75 years of age, recruited from the entire Sweden as our analytical study sample (Fig. 1). A detailed description of the data collection of SLOSH has been published elsewhere (Magnusson Hanson et al. 2008, 2015; Statistics Sweden 2014).

### Ethical approval

SLOSH has been approved by the Regional Research Ethics Board in Stockholm, and the present study has also received complementary approval by the Regional Research Ethics Board in Linköping #2014/355-31.

### Work-related stress measures

ICT demands were measured by a scale specifically developed for SLOSH, based on previous qualitative work by Johansson-Hidén et al. (2003). The scale was introduced by the following ingress in the questionnaire; “New technology and more flexible working conditions have changed working life for many people. Technology can be very helpful, but also contribute to new types of stress. Estimate to what extent you are stressed by…”. The ingress was accompanied with the following six items: “…too many calls and emails”, “…claims to be available on work-related issues during work hours”, “…claims to be available on work-related issues during leisure time”, “…claims to give immediate answers to emails and telephone calls that require a lot of work”, “…constantly being interrupted by the telephone and email” and “…computers and other equipment that fail to work properly”. The response options were rated on a Likert scale from 1 (I do not have access to this at work) to 5 (very much). The population mean score was calculated and used as cut-off values for high and low ICT demands (high ICT demands were defined as strictly above the median, and low ICT demands were defined as equal to or below the median).

Job strain was measured by the demand-control questionnaire (DCQ), comprising the dimensions of demands and control (Karasek and Theorell 1990). The dimension of demands was covered by five items, e.g. “Do you have to work very intensively?”. Control was covered by six items, e.g. “Do you have a choice in deciding how you do your work?”. The response options were rated on a Likert scale from 1 (no, hardly ever) to 4 (yes, often). The population mean response scores for the demands and control dimensions were computed and used as cut-off values for high or low scores of demands and control (“low demands” were defined as equal to or below the median, and “high demands” were defined as strictly above the median; “low control” was defined as strictly below the median, and “high control” was defined as equal to or above the median). “Job strain” was defined as a combination of high demands and low control and was compared with all other combinations of the demands and control dimensions.

ERI was measured by the short version of the ERI questionnaire, which comprised the dimensions of effort and reward (Siegrist 2013). Effort was covered by three items, e.g. “I have constant time pressure due to a heavy work load”. Reward was covered by seven items, e.g. “Considering all my efforts and achievements, I receive the respect and prestige I deserve at work”. The response options were rated on a Likert scale from 1 (strongly disagree) to 4 (strongly agree). ERI was defined as a combination of high effort and low reward, calculated by dividing the effort dimension with the reward dimension and using the ratio of 1 as cut-off value, with ratios strictly above 1 considered as ERI and ratios equal to or below 1 considered as no imbalance (Siegrist 2013).

Cronbach’s alpha was calculated on all included work-related stress dimensions on the present study sample, resulting in values between 0.65 and 0.85, where the value of 0.85 represented ICT demands.

### Self-rated health

Suboptimal self-rated health was analysed as outcome measure and measured by a one-item question; “How would you rate your general state of health?”. The response options were rated on a Likert scale from 1 (very good) to 5 (very bad). In the analyses, suboptimal self-rated health (defined as responding very bad, rather bad or neither good nor bad to the question) was contrasted to good self-rated health (defined as responding very good or rather good to the question).

### Covariates

Sex, age, SES, lifestyle factors (smoking and leisure time physical activity), BMI and other types of work-related stress were considered to be potential confounders or mediating factors. SES was analysed in the categories: low SES (unskilled, semiskilled and skilled workers), intermediate SES (assistant and intermediate non-manual workers) and high SES (employed and self-employed professionals, higher civil servants and executives), classified in line with Statistics Sweden’s manual of the socio-economic index (Statistics Sweden 1982). Smoking was analysed in the categories never smoker, ex-smoker and smoker. Physical leisure time activity was analysed in the categories: low/occasional physical activity and regular physical activity. BMI was calculated by self-reported weight in kilograms/height in squared metres and classified into four categories: underweight (<18.50), normal weight (18.50–24.99), overweight (25.00–29.99) and obesity (≥30.00) (World Health Organization 2015).

### Statistical analyses

Chi-square tests were conducted to analyse differences in proportions. ANOVAs were conducted to calculate differences between the mean values in different strata of SES. Spearman’s correlation coefficients were calculated to determine correlations between different continuous dimensions of work-related stress. Logistic regression analyses calculating odds ratios (OR) with 95 % confidence intervals (CI) were calculated to examine the association between dichotomised measures of work-related stress and suboptimal self-rated health. ICT demands, job strain and ERI were analysed separately in relation to suboptimal self-rated health by applying crude analyses and three different sequential regression models adjusted for age, sex and SES; age, sex, SES, lifestyle and BMI; and age, sex, SES, lifestyle, BMI and other types of work-related stress (e.g. job strain and ERI when analysing ICT demands). Alpha was set to 0.05. All analyses were carried out in the total study sample and stratified by SES. To test potential effect modification by SES, statistical interaction terms between the work-related stress measures and SES were included in the regression models. The software IBM SPSS Statistics 21 was used to calculate the results.

## Results

### Characteristics in participants with low, intermediate and high SES

Statistically significant differences in the proportion of ICT demands, job strain and ERI were observed between different SES strata (Table 1). ICT demands were most prevalent among participants with high SES (59.8 %), followed by participants with intermediate SES (54.9 %) and low SES (29.1 %). In contrast, job strain was most prevalent among participants with low SES (29.5 %), followed by participants with intermediate SES (19.1 %) and high SES (10.9 %). ERI was most prevalent among participants with intermediate SES (53.1 %), followed by participants with low SES (50.8 %) and high SES (46.6 %). Statistically significant differences in the prevalence of suboptimal self-rated health were observed, and suboptimal self-rated health was most prevalent among participants with low SES (24.8 %), followed by participants with intermediate SES (18.2 %) and high SES (16.7 %). Social gradients in physical activity, smoking and BMI were also observed (Table 1).

**Table 1:** Characteristics in the total study sample and in different SES strata

Characteristics	Total study sample *n* = 14,757 (100.9) *n* = 14,311 (100.0)	Low SES *n* = 4090 (28.6)	Intermediate SES *n* = 6928 (48.4)	High SES *n* = 3293 (23.0)	*p* value^a^
Age (cont.), mean (SD)	50.8 (10.0)	51.7 (10.0)	50.6 (10.0)	50.0 (10.1)	<0.001
Sex (cat. *n* (%))					
Men	6342 (43.0)	2095 (51.2)	2377 (34.3)	1595 (48.4)	<0.001
Women	8415 (57.0)	1995 (48.8)	4551 (65.7)	1698 (51.6)	
ICT demands (cat.), *n* (%)					
Low	7566 (51.3)	2900 (70.9)	3126 (45.1)	1324 (40.2)	<0.001
High	7191 (48.7)	1190 (29.1)	3802 (54.9)	1969 (59.8)	
ICT demands (cont.), mean (SD)	2.9 (0.7)	2.6 (0.7)	3.0 (0.7)	3.1 (0.7)	<0.001
DCQ (cat.), *n* (%)					
No strain	11,754 (80.0)	2871 (70.5)	5582 (80.9)	2926 (89.1)	<0.001
Job strain	2943 (20.0)	1200 (29.5)	1321 (19.1)	357 (10.9)	
Demands (cont.), mean (SD)	2.6 (0.7)	2.6 (0.8)	2.6 (0.6)	2.7 (0.6)	<0.001
Control (cont.), mean (sd)	3.1 (0.4)	2.9 (0.5)	3.1 (0.4)	3.3 (0.4)	<0.001
ERI (cat.), *n* (%)					
No ERI	7215 (49.3)	1996 (49.2)	3233 (46.9)	1747 (53.4)	<0.001
ERI	2427 (50.7)	2060 (50.8)	3655 (53.1)	1525 (46.6)	
Effort (cont.), mean (SD)	2.7 (0.7)	2.6 (0.8)	2.8 (0.7)	2.8 (0.7)	<0.001
Reward (cont.), mean (SD)	2.7 (0.8)	2.6 (0.9)	2.7 (0.8)	2.8 (0.5)	<0.001
Self-rated health (cat.), *n* (%)					
Suboptimal	2870 (19.6)	1004 (24.8)	1252 (18.2)	547 (16.7)	<0.001
Good	11,781 (80.4)	3048 (75.2)	5624 (81.8)	2734 (83.3)	
Physical activity (cat.), *n* (%)					
Low/occasional	7834 (53.4)	2497 (61.4)	3481 (50.5)	1593 (48.6)	<0.001
Regular	6846 (46.6)	1569 (38.6)	3416 (49.5)	1683 (51.4)	
Smoking (cat.), *n* (%)					
Never smoker	9830 (67.6)	2420 (60.2)	4721 (69.1)	2383 (73.4)	<0.001
Ex-smoker	3185 (21.9)	967 (24.1)	1480 (21.7)	647 (19.9)	
Smoker	1522 (10.5)	633 (15.7)	627 (9.2)	218 (6.7)	
BMI (cat.) *n* (%)					
<18.50	119 (0.8)	12 (0.3)	68 (1.0)	34 (1.0)	<0.001
18.50–24.99	6767 (46.8)	1537 (38.5)	3347 (49.2)	1700 (52.4)	
25.00–29.99	5607 (38.8)	1733 (43.4)	2503 (36.8)	1189 (36.7)	
≥30.00	1976 (13.7)	709 (17.8)	886 (13.0)	321 (9.9)	

The column percentage is presented in the parenthesis for categorical variables, e.g. there are 43.0 % men and 57.0 % women in the total study sample. The digit of *n* varies due to an internal attrition

^a^Chi-square test for comparison of proportions; ANOVA for comparisons of continuous variables

### Association between ICT demands and the dimensions of job strain and ERI

In the total study sample, the continuous measure of ICT demands was correlated with all the work-related stress dimensions of job strain and ERI (Table 2). Some of the correlations were low, but the strongest correlation was observed between ICT demands and effort (*r* = 0.51 *p* ≤ 0.001), followed by demands (*r* = 0.42; *p* ≤ 0.001).

**Table 2:** Spearman’s correlation coefficient between different work-related stress measures in the total study sample and in different SES strata

	ICT demands		Demands		Control		Effort	
*Total study sample*								
Demands	0.42**	(14,703)	–	–				
Control	0.11**	(14,745)	0.01	(14,703)	–	–		
Effort	0.51**	(14,703)	0.69**	(14,691)	0.02**	(14,699)	–	–
Reward	−0.08**	(14,646)	−0.30**	(14,637)	0.28**	(14,646)	−0.23**	(14,642)
*Low SES*								
Demands	0.29**	(4074)	–	–				
Control	0.15**	(4088)	−0.06**	(4074)	–	–		
Effort	0.40**	(4076)	0.69**	(4070)	0.01	(4074)	–	–
Reward	−0.10	(4057)	−0.37**	(4053)	0.27**	(4057)	−0.32**	(4056)
*Intermediate SES*								
Demands	0.47**	(6905)	–	–				
Control	−0.03*	(6924)	0.02	(6905)	–	–		
Effort	0.52**	(6904)	0.69**	(6901)	−0.01	(6904)	–	–
Reward	−0.16**	(6890)	−0.32**	(6887)	0.20**	(6890)	−0.27**	(6888)
*High SES*								
Demands	0.51**	(3284)	–	–				
Control	−0.07**	(3290)	−0.01	(3284)	–	–		
Effort	0.55**	(3285)	0.69**	(3283)	−0.02	(3284)	–	–
Reward	−0.17	(3272)	−0.26**	(3271)	0.24**	(3272)	−0.21**	(3272)

Values in the parenthesis represent *n* in each category. The digit of *n* varies due to an internal attrition

* *p* < 0.05; ** *p* < 0.01; *** *p* < 0.001

Statistically significant differences in the proportions of the dichotomised measure of ICT demands and job strain and ICT demands and ERI were observed (Table 3). Among participants with high SES, 80.7 % of those who reported job strain also reported high ICT demands. Among participants with intermediate SES, 75.2 % of those who reported job strain also reported high ICT demands, and among participants with low SES, only 36.3 % of those with job strain also reported high ICT demands. Concerning ERI, among participants with high SES, 77.8 % of those who reported ERI also reported high ICT demands, followed by 69.2 % among participants with intermediate SES and 40.0 % among participants with low SES (Table 3).

**Table 3:** Proportions of ICT demands and job strain or ICT demands and ERI in the total study sample and in different SES strata

ICT demands	DCQ			ERI		
	No strain	Job strain	*p* value^a^	No ERI	ERI	*p* value^a^
Total, *n* (%)						
Low	6346 (54.0)	1186 (40.3)	<0.001	4741 (65.7)	2754 (37.1)	<0.001
High	5408 (46.0)	1757 (59.7)		2474 (34.3)	4673 (62.9)	
SES, *n* (%)						
Low						
Low	2124 (74.0)	764 (63.7)	<0.001	1641 (82.2)	1235 (60.0)	<0.001
High	747 (26.0)	436 (36.3)		355 (17.8)	825 (40.0)	
Intermediate						
Low	2785 (49.9)	328 (24.8)	<0.001	1978 (61.2)	1125 (30.8)	<0.001
High	2797 (50.1)	993 (75.2)		1255 (38.8)	3530 (69.2)	
High						
Low	1249 (42.7)	69 (19.3)	<0.001	972 (55.6)	338 (22.2)	<0.001
High	1677 (57.3)	288 (80.7)		775 (44.4)	1187 (77.8)	

The column percentage is presented in the parenthesis for categorical variables, e.g. out of those in the total sample who reports job strain, 59.7 % also report high ICT demands. The digit of n varies due to an internal attrition

^a^Chi-square test for comparison of proportions

### Association between work-related stress measures and suboptimal self-rated health

ICT demands were associated with suboptimal self-rated health in crude analysis (OR 1.35 [CI 1.24–1.46]) and also after adjustment for age, sex, SES, lifestyle and BMI (OR 1.49 [CI 1.36–1.63]) (Table 4). When adding adjustment for job strain and ERI, the association between ICT demands and suboptimal self-rated health was attenuated but remained statistically significant (OR 1.18 [CI 1.07–1.30]). However, job strain (OR 1.93 [CI 1.74–2.14]) and ERI ((OR 2.15 [CI 1.95–2.35]), adjusted for age, sex, SES, lifestyle and BMI) showed fairly stronger associations with suboptimal self-rated health.

**Table 4:** Association between different measures of work-related stress and suboptimal self-rated health

	Crude		Adjusted for age, sex and SES		Adjusted for age, sex, SES, lifestyle and BMI		Adjusted for age, sex, SES, lifestyle, BMI and work-related stress measures	
	OR	CI 95 %	OR	CI 95 %	OR	CI 95 %	OR	CI 95 %
*Total study sample*								
Low ICT demands	1	(Ref)	1	(Ref)	1	(Ref)	1	(Ref)
High ICT demands	1.35	1.24–1.46	1.53	1.41–1.68	1.49	1.36–1.63	1.18	1.07–1.30
No strain	1	(Ref)	1	(Ref)	1	(Ref)	1	(Ref)
Job strain	2.04	1.86–2.24	1.92	1.75–2.12	1.93	1.74–2.14	1.50	1.35–1.68
No ERI	1	(Ref)	1	(Ref)	1	(Ref)	1	(Ref)
ERI	2.22	2.04–2.42	2.22	2.03–2.43	2.15	1.95–2.35	1.85	1.67–2.04
*Low SES*								
Low ICT demands	1	(Ref)	1	(Ref)	1	(Ref)	1	(Ref)
High ICT demands	1.44	1.24–1.68	1.42	1.21–1.65	1.39	1.18–1.63	1.11	0.94–1.33
No strain	1	(Ref)	1	(Ref)	1	(Ref)	1	(Ref)
Job strain	2.05	1.77–2.38	2.13	1.83–2.48	2.20	1.87–2.59	1.71	1.43–2.04
No ERI	1	(Ref)	1	(Ref)	1	(Ref)	1	(Ref)
ERI	2.17	1.87–2.51	2.24	1.93–2.61	2.24	1.91–2.63	1.81	1.51–2.17
*Intermediate SES*								
No ICT demands	1	(Ref)	1	(Ref)	1	(Ref)	1	(Ref)
High ICT demands	1.64	1.45–1.87	1.63	1.43–1.86	1.62	1.42–1.86	1.28	1.11–1.48
No strain	1	(Ref)	1	(Ref)	1	(Ref)	1	(Ref)
Job strain	1.86	1.62–2.15	1.87	1.62–2.16	1.86	1.59–2.16	1.42	1.21–1.67
No ERI	1	(Ref)	1	(Ref)	1	(Ref)	1	(Ref)
ERI	2.31	2.03–2.64	2.29	2.00–2.61	2.18	1.90–2.51	1.86	1.60–2.16
*High SES*								
Low ICT demands	1	(Ref)	1	(Ref)	1	(Ref)	1	(Ref)
High ICT demands	1.50	1.24–1.83	1.51	1.24–1.84	1.36	1.10–1.68	1.07	0.85–1.34
No strain	1	(Ref)	1	(Ref)	1	(Ref)	1	(Ref)
Job strain	1.76	1.36–2.29	1.71	1.31–2.23	1.60	1.20–2.13	1.35	1.01–1.81
No ERI	1	(Ref)	1	(Ref)	1	(Ref)	1	(Ref)
ERI	2.13	1.76–2.57	2.13	1.76–2.59	1.97	1.60–2.41	1.86	1.49–2.31

SES is only adjusted for in the analysis of the total study sample

Logistic regressions calculating the association between dichotomised measures of work-related stress and suboptimal self-rated health. Odds ratios and 95 % confidence intervals

When the analysis was stratified by SES, the association was somewhat stronger between ICT demands and suboptimal self-rated health among participants with intermediate SES (OR 1.62 [CI 1.42–1.86]), followed by participants with low SES (OR 1.39 [CI 1.18–1.63]) and high SES ((OR 1.36 [CI 1.10–1.68]), adjusted for age, sex, lifestyle and BMI) (Table 4). Similar and consistent patterns were observed in the crude and all adjusted analyses. However, test for statistical interaction between ICT demands and SES was not statistically significant in any of those models.

Concerning job strain, the association between job strain and suboptimal self-rated health was somewhat stronger among participants with low SES, followed by participants with intermediate SES and high SES (Table 4). Similar and consistent patterns were found in the crude and all adjusted analyses. Concerning ERI, the association between ERI and suboptimal self-rated health was rather similar in the different SES strata (Table 4). However, neither for job strain nor for ERI, the test for statistical interaction with SES was statistically significant.

## Discussion

The present study provides an overview of different types of work-related stress in modern working life and contributes to new knowledge about how ICT demands are associated with two types of work-related stress defined as job strain and ERI, and in association with suboptimal self-rated health, in general and in different SES strata. The results showed that ICT demands were more prevalent among participants with high and intermediate SES. ICT demands correlated statistically significant with the dimensions of job strain and effort-reward imbalance, especially with the demands and effort dimensions. Moreover, all the analysed work-related stress measures were associated with suboptimal self-rated health, but the association between ICT demands and suboptimal self-rated health was somewhat weaker than for job strain or ERI.

Even though the correlations in some cases were rather weak, statistically significant associations between ICT demands and other types of work-related stress were observed. This result is supported by previous findings that extensive ICT use of dysfunctional ICT hard- and software is associated with experience of stress (Chesley 2014; Day et al. 2012; Johansson-Hidén et al. 2003) as well as increased cortisol levels (Riedl et al. 2012). However, it should be noted that the correlations between ICT demands and the demands and effort dimensions in job strain and effort-reward imbalance scales may partly be due to a conceptual overlap, since the ICT demands scale asks the participants to rate whether they “are stressed” by the respective demands.

In line with other studies in occupational and public health (Hammig and Bauer 2013; Hoven and Siegrist 2013; Toivanen 2011), social gradients in the established work-related stress measures were observed. However, in the literature, little has been shown about the prevalence of ICT demands in different socio-economic strata. This study shows that ICT demands are more common among participants with high and intermediate SES, a result which deviates from the traditional socio-economic pattern of work-related stress. The reason why ICT demands were more prevalent in high and intermediate SES strata cannot be determined in the present study, but potential explanations are an overall higher ICT use, and an experience that many ICT-related tasks are added to the primary job duties in these groups. However, this may not be the only explanation, given that ICT demands are simply one part of the daily demands among gainfully employed people.

All the work-related stress measures used in the present study, including ICT demands, were associated with suboptimal self-rated health. Associations between job strain, ERI and suboptimal self-rated health have previously been observed in the literature (Hammig and Bauer 2013; Hoven and Siegrist 2013; Toivanen 2011), but the association between ICT demands and suboptimal self-rated health has not been examined before, to the best of our knowledge. The reason why ICT demands showed a weaker association with suboptimal self-rated health than job strain and ERI is unknown. However, one potential explanation could be that the ICT scale used in the present study only covers the demand dimension and not some other dimensions of interest, e.g. ICT-related recourses. A further developed ICT scale could be more sensitive and potentially show a stronger association with suboptimal self-rated health than the present ICT demands scale.

### Strengths and limitations

The present study contributes to an up-to-date picture of the work-related stress in general and in different SES strata in the modern working life, and new information about how ICT demands associate with other types of work-related stress and self-rated health. Generalisability of the results is strengthened by a relatively large sample size, including participants from both sexes, a wide range of ages, different SES strata and from different parts of Sweden, which embodies a rather representative cross section of the gainfully employed people in Sweden at the present time. The representativeness of the study sample is also, to some extent, supported by similar prevalence of background characteristics (e.g. self-rated health, lifestyle factors and BMI) as have been observed in other studies (Alvarez-Galvez et al. 2013; Padyab and Norberg 2014).

ICT demands were contrasted to work-related stress measured with the DCQ model and the short version of the ERI model, both measures that have been found valid and reliable, which strengthens the internal validity (Chungkham et al. 2013; Hokerberg et al. 2014; Leineweber et al. 2010). In addition, the internal reliability was strengthened by acceptable scores of Cronbach’s alpha at the work-related stress measures, not least ICT demands that showed a Cronbach’s alpha of 0.85.

The present study also has some limitations to consider. The ICT demands scale has not been validated at the time of writing. Even though this study provides some support that the concept of ICT demands is useful as a potential stressor of work-related stress, an actual validation study of the scale used is desired. Another concern with the ICT demands scale is that it does not provide a full explanation of why some people might experience high demands linked to some ICT-related working contexts. A further development and extension of the ICT scale, including a dimension that takes the individual’s resources regarding ICT use into account, is warranted, in order to get a more complete picture of ICT demands in relation to resources.

There are also some issues about using self-rated health as a health indicator. Measuring self-rated health by a one-item question provides an indication of the general health status of a person, but this measure can also be viewed as unspecific due to its association with a number of risk factors and diseases (Stenholm et al. 2014; Waller et al. 2015), and the present study does not provide information about specific health problems that contribute to poorer self-rated health.

Some matters concerning temporality, power, precision and generalisability should also be acknowledged. The temporality in the associations between work-related stress and suboptimal self-rated health cannot be established due to the cross-sectional design. Moreover, even though the power when analysing the total study sample is good, the power is lower in some of the SES stratified regression analyses, which affects the precision of the results. In addition, the influence of selection bias cannot be ruled out due to the somewhat low response rate, and the lower participation rate among men, younger people and people with low SES. This may limit the generalisability to the general working population. Hence, future studies based on other populations are needed to confirm the results.

## Conclusions

ICT demands are associated with job strain, ERI and suboptimal self-rated health and are common in people with intermediate and high SES. ICT demands should thus be acknowledged as a potential stressor of work-related stress in modern working life.

## Abbreviations

DCQ: Job demand-control questionnaire
ERI: Effort-reward imbalance
SES: Socio-economic status
BMI: Body mass index
OR: Odds ratio
CI: Confidence interval
